# Development of a biomarker prediction model for post-trauma multiple organ failure/dysfunction syndrome based on the blood transcriptome

**DOI:** 10.1186/s13613-024-01364-5

**Published:** 2024-08-28

**Authors:** Ivan Duran, Ankita Banerjee, Patrick J. Flaherty, Yok-Ai Que, Colleen M. Ryan, Laurence G. Rahme, Amy Tsurumi

**Affiliations:** 1 Department of Surgery, Massachusetts General Hospital and Harvard Medical School, 50 Blossom St., Their 340, Boston, MA 02114 USA; 2 Department of Mathematics and Statistics, University of Massachusetts at Amherst, Amherst, MA 01003 USA; 3 Department of Intensive Care Medicine, Inselspital, Bern University Hospital, University of Bern, Bern, Switzerland; 4 Shriners Hospitals for Children-Boston®, 51 Blossom St., Boston, MA 02114 USA; 5 Department of Microbiology and Immunology, Harvard Medical School, 77 Ave. Louis Pasteur, Boston, MA 02115 USA

**Keywords:** Organ failure, Trauma, Infections, Prediction, Personalized medicine, Machine learning, Biomarkers

## Abstract

**Background:**

Multiple organ failure/dysfunction syndrome (MOF/MODS) is a major cause of mortality and morbidity among severe trauma patients. Current clinical practices entail monitoring physiological measurements and applying clinical score systems to diagnose its onset. Instead, we aimed to develop an early prediction model for MOF outcome evaluated soon after traumatic injury by performing machine learning analysis of genome-wide transcriptome data from blood samples drawn within 24 h of traumatic injury. We then compared its performance to baseline injury severity scores and detection of infections.

**Methods:**

Buffy coat transcriptome and linked clinical datasets from blunt trauma patients from the Inflammation and the Host Response to Injury Study (“Glue Grant”) multi-center cohort were used. According to the inclusion/exclusion criteria, 141 adult (age ≥ 16 years old) blunt trauma patients (excluding penetrating) with early buffy coat (≤ 24 h since trauma injury) samples were analyzed, with 58 MOF-cases and 83 non-cases. We applied the Least Absolute Shrinkage and Selection Operator (LASSO) and eXtreme Gradient Boosting (XGBoost) algorithms to select features and develop models for MOF early outcome prediction.

**Results:**

The LASSO model included 18 transcripts (AUROC [95% CI]: 0.938 [0.890–0.987] (training) and 0.833 [0.699–0.967] (test)), and the XGBoost model included 41 transcripts (0.999 [0.997–1.000] (training) and 0.907 [0.816–0.998] (test)). There were 16 overlapping transcripts comparing the two panels (0.935 [0.884–0.985] (training) and 0.836 [0.703–0.968] (test)). The biomarker models notably outperformed models based on injury severity scores and sex, which we found to be significantly associated with MOF (APACHEII + sex—0.649 [0.537–0.762] (training) and 0.493 [0.301–0.685] (test); ISS + sex—0.630 [0.516–0.744] (training) and 0.482 [0.293–0.670] (test); NISS + sex—0.651 [0.540–0.763] (training) and 0.525 [0.335–0.714] (test)).

**Conclusions:**

The accurate assessment of MOF from blood samples immediately after trauma is expected to aid in improving clinical decision-making and may contribute to reduced morbidity, mortality and healthcare costs. Moreover, understanding the molecular mechanisms involving the transcripts identified as important for MOF prediction may eventually aid in developing novel interventions.

**Supplementary Information:**

The online version contains supplementary material available at 10.1186/s13613-024-01364-5.

## Background

Trauma is among the leading causes of morbidity, mortality, increased length of stay and healthcare costs [1–3]. Multiple organ failure/dysfunction syndrome (MOF/MODS) is one major adverse outcome with a high incidence among trauma patients [4–7], who experience acute and prolonged immune dysregulation [8–15] and a high incidence of infections [16–18]. MOF/MODS is identified as a significant source of mortality and resource consumption in this population [19, 20], suggesting that timely detection of post-trauma MOF/MODS soon after injury to achieve appropriate and efficient delivery of early preventative and management measures is expected to improve patient outcomes and mitigate healthcare costs. Moreover, identifying novel clinical factors and molecular mechanisms associated with MOF to elucidate mechanisms underlying its development is expected to be impactful.

Current clinical practices for diagnosing patients entail monitoring MOF/MODS-specific physiological score systems such as the Denver [21], Marshall multiple organ dysfunction score (MODS) [22] or sequential organ failure assessment (SOFA) [23] scores to detect its onset. Various studies have assessed the ability of common injury severity scores computed soon after admission, including the Acute Physiology and Chronic Health Evaluation (APACHE) II [24], Injury Severity Score (ISS) [25] and New Injury Severity Score (NISS) [26] as predictors of trauma-related MOF/MODS [27–30] and infections [31, 32]. However, these scores are limited in accuracy and timeliness of outcome detection and are based on gross clinical measures that do not account for individual molecular responses to injury. Indeed, it has been reported previously that injury severity scores and immune responses are not consistent in their ability to predict clinical outcomes post-trauma [33], and it has been noted that novel methods based on molecular biomarkers are needed to improve monitoring MODS [34].

Given that trauma patients are at especially high risk for MOF/MODS, developing novel biomarkers for accurate prediction is imperative. Novel machine learning (ML) algorithms provide immense potential to support the implementation of personalized medicine approaches using genome-wide data to ameliorate deficiencies of current practices involving clinical scores generally across all patients. Injury severity scores are limited as a method for populational assessment, rather than a valid approach for prognostication at the individual level, which gene expression signatures would allow. Such an ML-based approach is expected to maximize the information obtained from each patient and aid in developing accurate prediction methods to improve clinical decision-making, enhance resource allocation and augment the quality and cost-effectiveness of patient care [35–37]. It has been noted that biomarkers to improve critical care is needed and that additional studies to determine which combinations of biomarkers can give optimum results are of immense interest [38].

Studies among trauma and burn patients using ML analysis of blood transcriptomic data to develop biomarker panels for the early prediction of infections have consistently shown that they significantly outperformed various injury severity scores [39–41]. Moreover, the advantage of the molecular profiling approach is highlighted by the uncovering of novel mechanisms. These studies suggest that applying ML to early blood transcriptomic data is likely a feasible method to develop prediction models for adverse post-trauma outcomes, including MOF/MODS, that are more accurate than clinical scores and aid in elucidating molecular factors involved.

Although there are previous studies that have leveraged transcriptomic data collected in trauma centers to discover transcripts associated with MODS [14], sepsis [42–44] and other poor outcomes post-injury [45], they were aimed at identifying differentially regulated transcripts rather than developing early prediction models. Another study that developed MOF prediction models evaluated common cytokines [46], rather than employing an unbiased ML approach. One study used the Least Absolute Shrinkage and Selection Operator (LASSO) and Elastic Net (EN) ML algorithms and identified decreased CD62L and CD63 neutrophil expression and CD63 monocyte expression as predictors of MODS, and showed improvement in performance over NISS [47]. Taken together, no previous study has applied ML analysis of genome-wide transcriptome data from early blood samples to develop and validate prediction models for MOF/MODS and make comparisons with different common injury severity scores. Moreover, in addition to the LASSO and EN penalized regression methods, more recently developed algorithms including eXtreme Gradient Boosting (XGBoost) [48] has yet to be used to develop prediction models for trauma-related outcomes. The SHapely Additive exPlanations (SHAP) [49, 50] scores can be determined to evaluate which features in the model contribute to the outcome prediction, making XGBoost more interpretable, highlighting its advantage. Thus, we employed both LASSO and XGBoost, which is novel and expected to improve the development of prediction models relevant to post-trauma outcomes.

We developed highly accurate early prediction methods for post-trauma MOF outcome, based on genome-wide transcriptomic data collected from early blood samples collected within 24 h of injury. This approach is expected to significantly improve the accuracy of early identification of trauma patients at risk of MOF using blood samples collected at triage for implementing risk stratification strategies to help improve patient outcomes. Potential early interventions for patients found to be at increased risk of MOF may include immediate admission to the Intensive Care Unit (ICU) rather than the Step-Down Unit, more aggressive hemodynamic and culture monitoring, improved resuscitation precision by implementing higher levels of monitoring and more aggressive drainage of infections. MOF risk evaluation may also be included as a factor among others for borderline resuscitation decisions and providing information to the patients’ family. Early MOF risk evaluation may also be included among factors for Crisis Standards of Care or military triage. MOF risk assessment is also expected to be advantageous for standardizing patient group selection in clinical research. Identifying novel molecular markers of MOF is also expected to enhance understanding of underlying mechanisms, which may aid in improving preventive approaches and therapeutics development.

## Methods

### Study design and population

Patient clinical and transcriptomic data were obtained from the Glue Grant (“Inflammation and Host Response to Injury”) cohort [51], a multi-center cohort that enrolled patients at US Level 1 trauma centers between 2003 and 2009. The sample collection/storage and data generation were performed by the Glue Grant Consortium, and the permission for the access and secondary analysis of de-identified data was obtained from the Massachusetts General Hospital Institutional Review Board (MGH IRB protocol 2002P001743).

### Inclusion/exclusion criteria

**Fig. 1:**
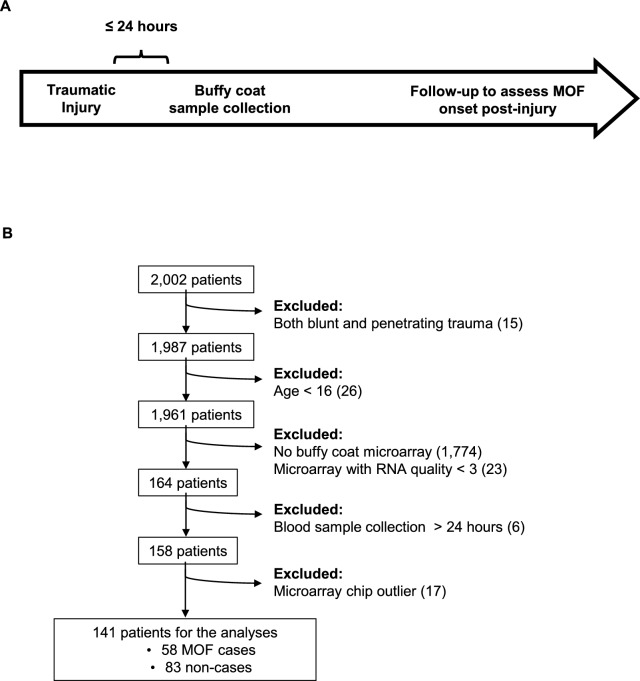
Description of the patient population and study design. **A** Schematic of the study design and timing of the blood sample collection, and **B** patients who were included/excluded in the study

Among the 2,002 patients in the dataset we obtained from the Glue Grant, 141 patients were included in the analyses. Our inclusion/exclusion criteria were as follows: adults aged ≥ 16 years old (excluding those < 16 years old) who sustained blunt trauma (excluding penetrating injury only or blunt with penetrating injury) with buffy coat collected early, within ≤ 24 h since trauma injury (excluding those without buffy coat collection or those collected after 24 h) and transcriptome data of high RNA quality of ≥ 3 out of 4 (excluding those with RNA quality below 3 and removing chips identified as outliers) (Fig. 1A, B). Where patients had multiple microarrays consistent with the inclusion criteria, the earliest timepoint was used. There were 58 MOF cases and 83 non-cases in total included in the study.

### Study variables

Clinical scores (APACHEII, ISS, NISS, Denver, Marshall) were recorded by participating institutions according to the guidelines outlined by the Glue Grant Consortium. Body mass index (BMI) was calculated from recorded height and weight (weight in kilograms/height in meters^2^). Patients were assigned to MOF-cases and non-cases, according to the Glue Grant Study’s recorded MOF onset day, indicated as using the criteria of Marshall score without the Glasgow Coma Scale ≥ 6.

### Software and packages

R version 4.3.2 was used for the analyses.

### Baseline characteristics calculations

For the analyses, MOF-cases (according to the Glue Grant Study’s criteria) were compared with non-cases, and the same criteria were used to develop the outcome prediction model. Baseline characteristics are reported means ± standard deviation (SD), or total numbers with proportions (%), as indicated in the legend (Table 1). Means between MOF-cases versus non-cases were compared by the unpaired equal variance two-tailed t-test. For comparing proportions, the Chi-square test was used for all expected values of 5 or greater, or Fisher’s exact test for an expected value below 5.

**Table 1:** Baseline characteristics of the study population

	All patients (N = 141)	MOF cases (n = 58)	Non-cases (n = 83)	*p-value*
Demographics				
Age	33.9 ± 11.0	33.1 ± 10.5	34.5 ± 11.3	0.468^a^
Sex: Female Male	53 (37.6%) 88 (62.4%)	14 (24.1%) 44 (75.9%)	39 (47.0%) 44 (53.0%)	0.010^b^
BMI	28.8 ± 6.8	28.1 ± 5.7	29.9 ± 8.0	0.131^a^
Severity Scores				
APACHE II	27.8 ± 5.7	28.9 ± 5.7	27.1 ± 5.7	0.073^a^
ISS	31.7 ± 13.8	34.3 ± 12.8	29.9 ± 14.3	0.058^a^
NISS	36.6 ± 13.1	38.5 ± 12.1	35.3 ± 13.7	0.155^a^
MOF outcome				
Days to MOF onset since injury	*NA*	3.2 ± 2.3	*NA*	–
Infections				
Overall, on any day during follow-up	90 (63.8%)	50 (86.2%)	40 (48.2%)	< 0.001^b^
First recorded before or on MOF onset day	*NA*	13 (22.4%)	*NA*	–
First recorded after MOF onset day	*NA*	37 (63.8%)	*NA*	–
Outcomes				
Mortality	7 (5.0%)	7 (12.1%)	0 (0%)	0.002^c^
Days in the ICU since injury	13.2 ± 10.0	19.7 ± 10.8	8.7 ± 6.5	< 0.001^a^
Days on ventilator since injury	10.0 ± 8.6	15.9 ± 9.3	5.9 ± 5.0	< 0.001^a^
Discharge day since injury	25.4 ± 17.4	32.3 ± 19.4	20.7 ± 14.0	< 0.001^a^

Comparisons are made between MOF cases (as defined by the Glue Grant Study) versus non-cases. Mean ± SD for continuous variables or n (%) for categorical variables are reported. P-value calculations are indicated as: ^a^Unpaired equal variance two-tailed t-test, ^b^Chi-square test, or ^c^Fisher’s Exact two-tailed test. *ICU days and discharge day since injury were calculated only among survivors

### Microarray analysis

The GCRMA [52] package (version 2.74.0) was used to process CEL files to normalized log_2_ expression values of probe sets. The arrayQualityMetrics [53] package (version 3.58.0) and principal components analysis with the factoextra [54] package (version 1.0.7) were used to remove outlier chips. Internal control and low abundance probe sets (*i.e.,* log_2_ expression value < 3 across all samples) were filtered, reducing the number of probe sets from 54,675 to 28,888 for the subsequent analyses. The limma [55] package (version 3.57.10) was used to calculate the log_2_ fold change values and false discovery rate (FDR)-adjusted p-values to compare MOF-cases versus non-cases. The top 500 most variable probe sets that also showed at least 1.2-fold change (105 probe sets) were used for the subsequent biomarker development.

### Machine learning prediction model development

The data was randomly split into 70% training (n = 100) and 30% test (n = 41) sets using the Caret [56] package (version 6.0.94). The Glmnet [57] package (version 4.1.8) was used to implement the least absolute shrinkage and selection operator (LASSO) regression to select probe sets that were predictive of MOF. The expression levels were standardized with the training set as the reference, using the Caret [56] package. The penalty weight, lambda (λ) that minimized the deviance was identified by performing tenfold cross-validation (CV) with 100 repeats. Probe sets were selected according to the hyperparameter values of ⍺ = 1 and λ = 0.0395, which yielded 19 probe sets, mapping to 18 transcripts. A multivariable logistic regression model was constructed for the outcome of MOF onset and the maximum likelihood coefficient estimates were obtained for the model with the 19 probe set predictors selected with LASSO.

To develop the eXtreme Gradient Boosting (XGBoost) model, Bayesian optimization was performed with tenfold CV on the training set to determine the hyperparameter values that maximized the mean test AUROC, using the ParBayesianOptimization [58] package (version 1.2.6) (learning rate = 0.146, maximum depth of a tree = 4, gamma = 1.519, minimum child weight = 2.800, subsample ratio = 0.851, column sample ratio = 0.971, L1 regularization = 0, L2 regularization = 2.829, number of boosting rounds = 45). The SHapely Additive exPlanations (SHAP) scores were found for each probe set with the SHAPforXGBoost [59] package (version 0.1.3), which yielded 42 probe sets, mapping to 41 transcripts with mean SHAP score above 0.

For identifying overlapping transcripts, mapped transcript names between those identified with LASSO and XGBoost were compared, and a Venn diagram was drawn using the ggvenn [60] package (version 0.1.10). From the LASSO model, the two probe sets mapping to the transcripts that were not included in the XGBoost model were removed, and a multivariable logistic regression model was constructed for the outcome of MOF onset and the maximum likelihood coefficient estimates were obtained. Multivariable logistic regression models were also constructed with various injury severity scores (APACHEII, ISS, NISS) and sex. All the models above were initially constructed in the training set and then evaluated in the test set.

The area under the receiver operating characteristic curve (AUROC) with DeLong 95% confidence intervals was calculated using the pROC [61] package (version 1.18.5). The sensitivity, specificity, positive predictive value (PPV) and negative predictive value (NPV) were calculated using the epiR [62] package (version 2.0.66).

### Functional assessment

Gene ontology (GO) and Kyoto Encyclopedia of Genes and Genomes (KEGG) pathway enrichment analyses were conducted for this panel of probe sets using the pathfindR [63] package (version 2.3.0). Terms with at least two genes included where plotted using the ggplot2 [64] package (version 3.4.4). A network plot was constructed using the GeneMANIA Cytoscape plug-in [65].

## Results

### Patient demographics and baseline characteristics show significantly higher injury severity scores and proportion of males among MOF-cases

Baseline demographic and injury characteristics, as well as clinical outcomes of the 141 blunt trauma patients included in the study (Fig. 1A, B) are presented in Table 1. Motor vehicle collisions were the most frequent injury mechanisms, and no significant differences in injury types and characteristics were found between MOF-cases and non-cases (Supplementary Table S1). The overall study population consisted of patients with a mean age of 33.9 ± 11.0 years old, with 37.6% females and 62.4% males. Age was not significantly different between MOF-cases and non-cases. However, the relative proportion of males was significantly higher for MOF-cases compared to non-cases (75.9% male versus 24.1% female MOF-cases and 53.0% male versus 47.0% female non-cases, p = 0.010). Baseline injury severity scores generally tended to be higher for MOF-cases compared to non-cases, comparing APACHEII (28.9 ± 5.7 for MOF-cases versus 27.1 ± 5.7 for non-cases, p = 0.073), ISS (34.3 ± 12.8 for MOF-cases versus 29.9 ± 14.3 for non-cases, p = 0.058) and NISS (38.5 ± 12.1 for MOF-cases versus 35.3 ± 13.7 for non-cases, p = 0.155). The average day to MOF diagnosis since injury among cases was 3.2 ± 2.3 days. On average, the maximum organ-specific MOF scores recorded were highest for the central nervous system score, followed by cardio score, respiratory score, renal score, hepatic score and finally, hematologic score (Supplementary Table S2).

### MOF is associated with worse patient outcomes

There were seven total patients who did not survive, who were all among the MOF-cases (12.1% among MOF-cases vs. 0% among non-cases, p = 0.002) (Table 1). The main causes of death for these patients were attributed to MOF, sepsis, shock, head injury, hypoxia and brain death (Table 2). MOF-cases, compared to the non-cases, also had significantly longer days in the ICU (19.7 ± 10.8 among MOF-cases vs. 8.7 ± 6.5 among non-cases, p < 0.001), on the ventilator (15.9 ± 9.3 among MOF-cases vs. 5.9 ± 5.0 among non-cases, p < 0.001) and discharge day since injury (32.3 ± 19.4 among MOF-cases vs. 20.7 ± 14.0 among non-cases, p < 0.001).

**Table 2:** Causes of death among non-survivors

MOF case	Death day since injury	Primary cause of death	Secondary cause of death
Yes	8	Multiple Organ Failure	–
Yes	9	Hypovolemic shock	Pulmonary Embolism
Yes	10	Multiple Organ Failure	–
Yes	10	Sepsis	Multiple Organ Failure
Yes	11	Severe Head Injury (Trauma only)	Cardiac Dysfunction
Yes	20	Hypoxia	–
Yes	24	Brain Death	–

### Infections were significantly increased among MOF-cases, and mostly occurred after MOF, rather than before

Among all patients, 63.8% had a record of infections during follow-up, regardless of MOF development (Table 1). Among MOF-cases, 86.2% had an incidence of infection at any time during follow-up, which was significantly higher than 48.2% among non-cases (p < 0.001). When assessing the timing of infection relative to MOF among cases, 22.4% were found to have the first record of an infection before or on the day of MOF diagnosis (n = 6 within 7 days and n = 7 on the same day), whereas 63.8% of patients had the first record of an infection after the MOF onset date (n = 23 at 1–7 days, n = 9 at 8–14 and n = 5 at 15–26 days) (Table 1, Supplementary Table S3). Overall, among MOF-cases, pneumonia (75.0%) was the most common infection type, followed by surgical site infections (42.3%), bloodstream infections (40.4%) and urinary tract infections (23.1%); similarly, among the non-cases, pneumonia (42.5%) was the most common, then surgical site and urinary infections (both 35.0%), then bloodstream infections (22.5%) (Table 3). Comparing infection types detected before and after MOF onset, pneumonia incidence was similar before and after MOF (69.2% before vs. 64.6% after), whereas for other infections, the proportion was larger after MOF onset.

**Table 3:** Microorganisms and infection types detected (NOS: not otherwise specified)

	Infections among MOF-cases (n = 50)	Infections before or on MOF onset day (n = 13)	Infections after MOF (n = 46*)	Infections among non-cases (n = 40)
Infection type				
Pneumonia	37 (75.0%)	9 (69.2%)	29 (64.6%)	17 (42.5%)
Surgical site infection	20 (42.3%)	2 (15.4%)	19 (43.8%)	14 (35.0%)
Bloodstream infection (including catheter-related)	19 (40.4%)	1 (7.7%)	18 (41.7%)	9 (22.5%)
Urinary tract infection	11 (23.1%)	1 (7.7%)	10 (22.9%)	14 (35.0%)
Other (Empyema, Pseudomembranous colitis, other)	9 (17.3%)	1 (7.7%)	8 (12.5%)	6 (15.0%)
Organism				
*Staphylococcus aureus*	14 (30.8%)	2 (15.4%)	13 (31.3%)	10 (25.0%)
*Enterobacter species*	13 (26.9%)	4 (30.8%)	10 (22.9%)	9 (22.5%)
*Acinetobacter*	14 (26.9%)	1 (7.7%)	14 (29.2%)	4 (10.0%)
*Enterococcus*	10 (21.2%)	1 (7.7%)	9 (20.8%)	5 (12.5%)
*Haemophilus influenza*	8 (15.4%)	2 (15.4%)	6 (12.5%)	1 (2.5%)
*Pseudomonas aeruginosa*	6 (13.5%)	1 (7.7%)	6 (14.6%)	6 (15.0%)
*E. coli*	4 (7.7%)	2 (15.4%)	2 (4.2%)	4 (10.0%)
*Klebsiella pneumoniae*	2 (7.7%)	0 (0%)	2 (8.3%)	0 (0%)
*Serratia marcescens*	3 (5.8%)	0 (0%)	3 (6.3%)	0 (0%)
*Coagulase-negative staphylococci*	3 (5.8%)	0 (0%)	3 (6.3%)	7 (17.5%)
*Bacteroides* species	2 (5.8%)	0 (0%)	2 (6.3%)	1 (2.5%)
Other gram negatives (*Neisseria*, *Stenotrophomonas*, *Proteus*, NOS)	9 (19.2%)	0 (0%)	9 (20.8%)	5 (12.5%)
Other gram positives (*Steptococci* species, *Clostridium* species, NOS)	7 (13.5%)	2 (23.1%)	4 (8.3%)	8 (20.0%)
Fungi (*Candida* species, *Aspergillus*, NOS)	6 (13.5%)	0 (0%)	6 (14.6%)	4 (10.0%)
Unknown	3 (5.8%)	0 (0%)	3 (6.3%)	3 (7.5%)
Polymicrobial^#^ (more than one organism)	30 (60.0%)	8 (61.5%)	30 (65.2%)	17 (42.5%)

*Patients with infections recorded after their MOF onset day may also include those who had the first infection recorded beforehand. ^#^Polymicrobial indicates patients with a record of more than one organism listed above—they are also represented in the calculations for each organism separately

The top three microorganisms detected overall among MOF-cases were *Staphylococcus aureus* (30.8%), *Enterobacter* species (26.9%) and *Acinetobacter* (26.9%), compared to non-cases with *Staphylococcus aureus* (24.4%), *Enterobacter* species (22.0%) and *Coagulase-negative staphylococci* (17.1%) (Table 2). Species with a higher proportion detected in infections before or on MOF onset day versus after included *Enterobacter* species (30.8% before or on MOF onset day vs. 22.9% after), *Haemophilus influenza* (15.4% before or on MOF onset day vs. 12.5% after), *E.coli* (15.4% before or on MOF onset day vs. 4.2% after) and other gram positives (23.1% before or on MOF onset day vs. 8.3% after) (Table 3). Pathogens not detected before or on MOF onset day, but only detected in infections after or among non-cases, included *Klebsiella pneumoniae*, *Serretia marcescens*, *Coagulase-negative staphylococci*, *Bacterioides*, other gram negatives and fungi species (Table 2). Polymicrobial infections were detected more frequently among MOF cases (60.0%) compared to non-cases (42.5%) (Table 3).

### The predictive biomarker model significantly improved early MOF detection compared to the injury severity models

**Fig. 2:**
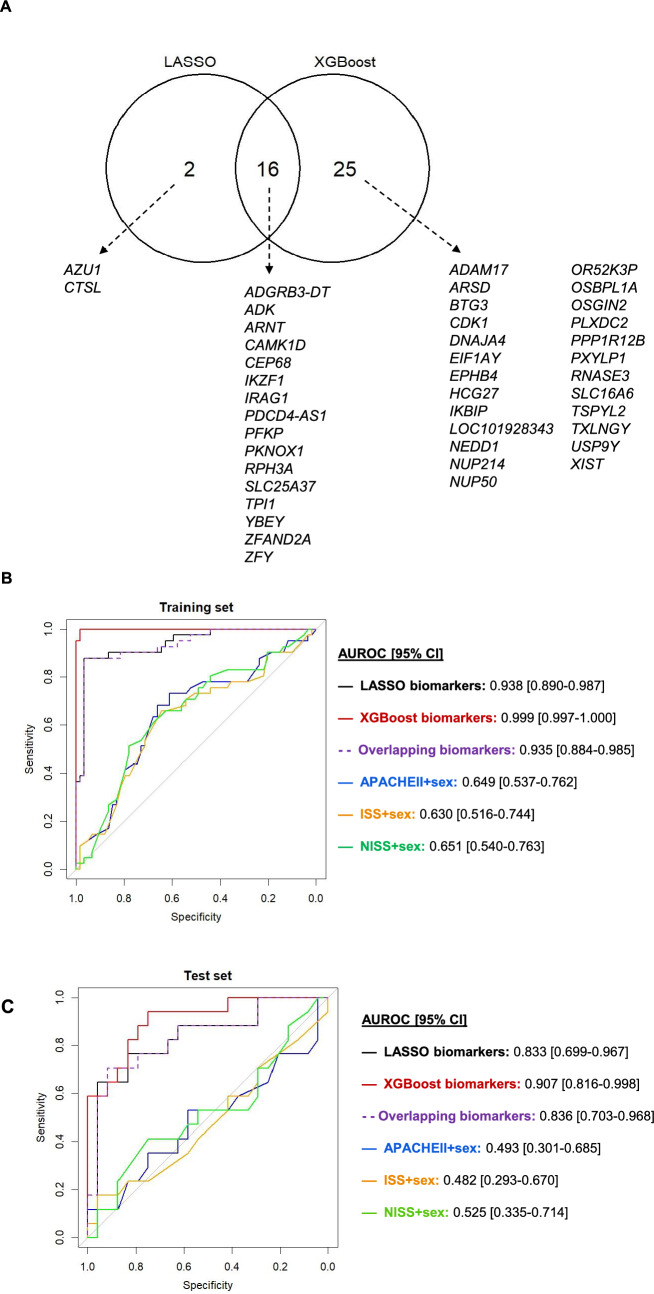

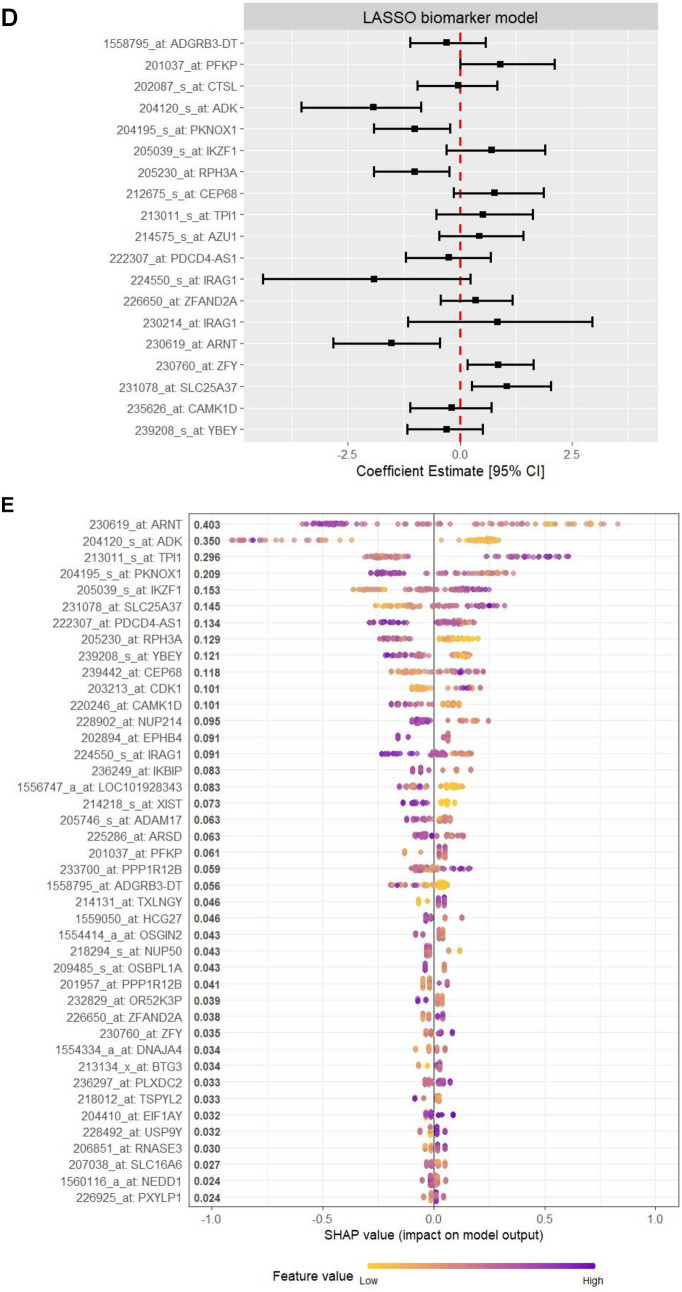

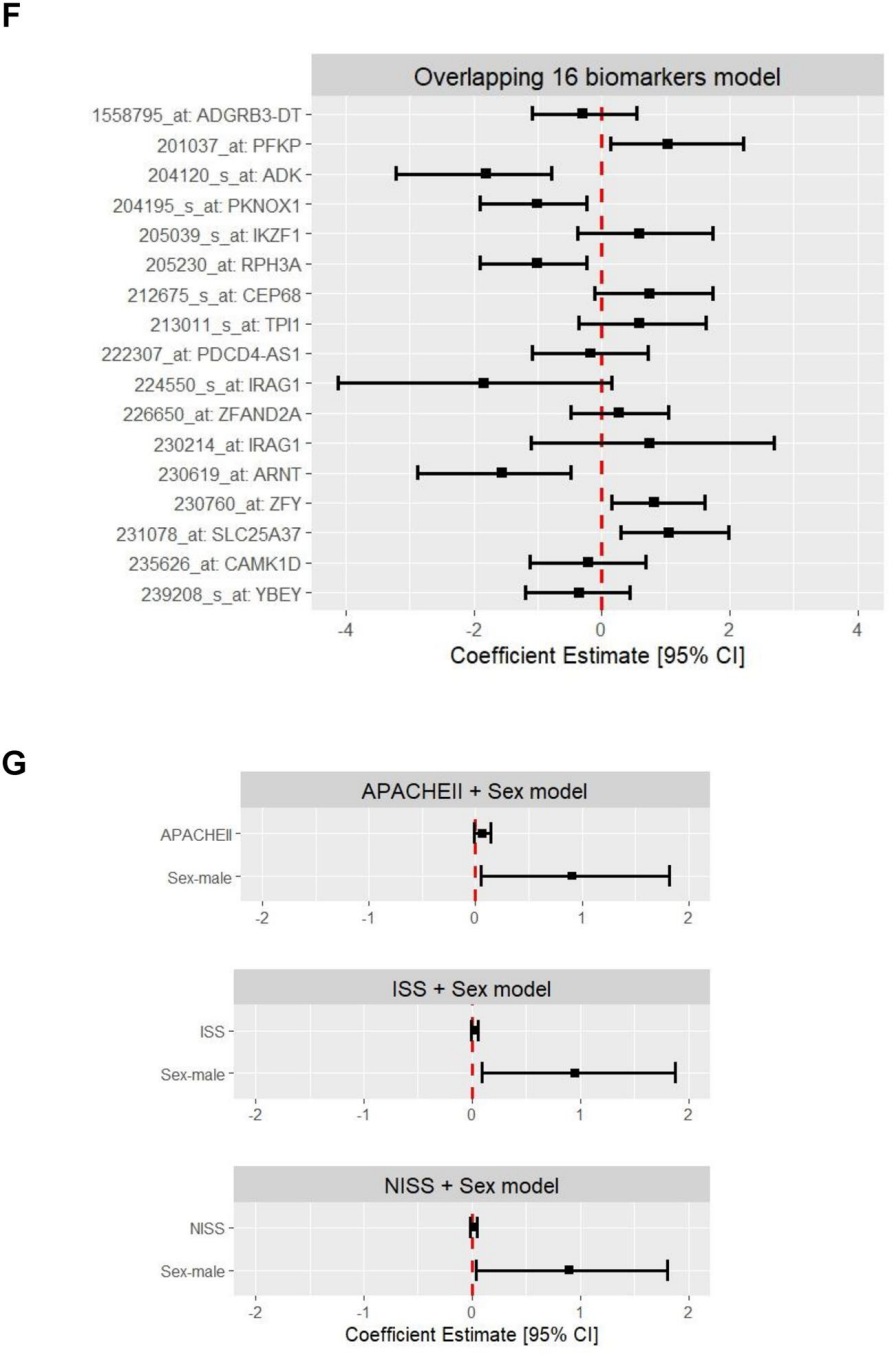
Description of the predictive biomarkers identified and comparisons of the performance of the various MOF outcome prediction models. **A** Summary of the transcript names that were unique or overlapping between the LASSO and XGBoost models. ROC curves and AUROC [95% CI] of each of the models constructed (LASSO biomarker panel with 19 probe sets mapping to 18 transcripts; XGBoost biomarker panel model with 42 probe sets mapping to 41 transcripts; overlapping 16 transcript panel (*i.e.* the LASSO panel minus the probe sets mapping to the two transcripts that were not included in the XGBoost panel); APACHEII + sex; ISS + sex; and NISS + sex), where results are shown for the models developed in the **(B)** training set, and then applied to the **(C)** test set. **(D)** The coefficient estimates for the LASSO model, **(E)** mean SHAP scores for the XGBoost model, **(F)** coefficient estimates for the overlapping transcripts model, and **(E)** coefficient estimates for the different clinical models are shown

The model developed using LASSO feature selection, included 19 probe sets, mapping to 18 transcripts and the XGBoost model identified 42 probe sets, mapping to 41 transcripts as being important for MOF outcome prediction. There were 16 overlapping transcripts in the LASSO and XGBoost models, suggesting their importance, and a total of 43 transcripts were identified across both models (Fig. 2A). A review of previous literature found that many of the 16 transcripts found by both LASSO and XGBoost have been implicated in traumatic injuries and organ failure/dysfunction, or immune cell functions (Table 4). The model developed using LASSO showed accurate MOF outcome prediction (AUROC [95% CI]: 0.938 [0.890–0.987] in the training set and 0.833 [0.699–0.967] in the test set) (Fig. 2B–D; Supplementary Table S4, S5), and the XGBoost model showed exceptional prediction performance (0.999 [0.997–1.000] in the training set and 0.907 [0.816–0.998] in the test set) (Fig. 2B, C, E; Supplementary Table S4). We further evaluated a simpler multivariable logistic regression model based on the 16 overlapping transcripts and found its performance to be similar to that of the model based on LASSO (0.935 [0.884–0.985] in the training set and 0.836 [0.703–0.968] in the test set) (Fig. 2B, C, F; Supplementary Table S4, S5). The biomarker-based models were notably better performing compared to models based on clinical variables of injury severity scores and sex (APACHEII + sex—0.649 [0.537–0.762] in the training set and 0.493 [0.301–0.685] in the test set; ISS + sex—0.630 [0.537–0.744] in the training set and 0.482 [0.293–0.670] in the test set; NISS + sex—0.651 [0.540–0.763] in the training set and 0.525 [0.335–0.714] in the test set), each showing the lack of ability to predict MOF (Fig. 2B, C, G; Supplementary Table S5).

**Table 4:** Previous literature on the overlapping 16 transcripts with their potential relevance to MOF

Implicated in traumatic injuries and organ failure/dysfunction	
* Adenosine Kinase* (*ADK*)	• Potential target to promote neuroprotection—in mice, its inhibition enhanced neural stem cell proliferation following traumatic brain injury [83] • In mice ischemic stroke models, its overexpression promoted stroke-induced brain injury, [84] while its downregulation was found to be protective [85] • Overexpressed as a result of vascular pro-inflammatory response, and its knockdown was found to increase adenosine levels and reduce endothelial inflammation [86]
* Aryl hydrocarbon receptor nuclear translocator* (*ARNT*) / *Hypoxia-Inducible Factor 1 Beta* (*HIF1B*))	• Forms heterodimers with other HIFs to regulate target genes with hypoxia-response elements (HREs), including during ischemic heart failure, when it was found to promote endothelial barrier integrity and vascular dysfunction prevention [87] • HIF signaling is induced in response to traumatic injuries [88] and severe burn injury-related kidney injury [89]
* Calcium/Calmodulin Dependent Protein Kinase 1D* (*CAMK1D*)	• Upregulated upon mechanical peripheral nerve injury and found to be important for dorsal root ganglion (DRG) neuron regeneration [90]
* Rabphilin 3A* (*RPH3A*)	• Upregulated in astrocytes and downregulated in neurons; identified as a neuroprotective response in a rat cerebral ischemia‐reperfusion injury model [91]
* Solute carrier family 25 member 37* (*SLC25A37*)	• Found among genes related to iron transport that were significantly upregulated in blood early after traumatic injury [92]
* Zinc finger, AN1-type domain 2A* (*ZFAND2A*)	• A marker for acute kidney ischemia–reperfusion injury in a rat model [93]
Implicated in immune responses	
* IKAROS family zinc finger 1* (*IKZF1*)	• Various mutations have been associated with misregulated lymphocyte and hematopoietic stem cell composition [94, 95]; and hematologic abnormalities and autoimmune diseases [96]
* Phosphofructokinase, Platelet Type* (*PFKP*)	• A well-established glycolysis regulator, which is downregulated by sirtuin 2 (SIRT2) and involved in reduced macrophage phagocytosis resulting from acute ethanol exposure [97] • Found to play an immune regulatory role by promoting glycolysis in different types of cancers [98–101] and in autoimmune diseases [102]
* PBX/Knotted 1 Homeobox 1* (*PKNOX1*)	• In cardiac and adipose tissues, found to promote the proinflammatory M1 macrophage phenotype and a direct target of downregulation by various micro-RNAs (miRs) that regulate M2 macrophage polarization [103]
* Triosephosphate Isomerase 1* (*TPI1*)	• Upregulated in lung adenocarcinoma and squamous cell carcinoma, where it promotes immune cell infiltration [104]
Potential link to MOF unknown	
* Adhesion G protein-coupled receptor B3 divergent transcript* (*ADGRB3*-*DT*)	
* Centrosomal Protein 68* (*CEP68*)	
* Inositol 1,4,5-triphosphate receptor associated 1* (*IRAG1*)	
* PDCD4 Antisense RNA 1* (*PDCD4-AS1*)	
* YbeY Metalloendoribonuclease* (*YBEY*)	
* Zinc Finger Protein Y-Linked* (*ZFY*)	

The sensitivity, specificity, positive predictive value (PPV) and negative predictive value (NPV) were also notably high for the biomarker prediction model (LASSO model—sensitivity 0.878, specificity 0.949, PPV 0.923 and NPV 0.918 in the training set, and sensitivity 0.765, specificity 0.750, PPV 0.684 and NPV 0.818 in the test set; XGBoost model—sensitivity 0.951, specificity 1.000, PPV 1.000 and NPV 0.967 in the training set, and sensitivity 0.833, specificity 0.706, PPV 0.800 and NPV 0.750 in the test set; overlapping biomarkers model—sensitivity 0.878, specificity 0.932, PPV 0.900 and NPV 0.917 in the training set, and sensitivity 0.765, specificity 0.750, PPV 0.684 and NPV 0.818 in the test set), as compared to the other models based on clinical variables (Table 5). Overall, the XGBoost model showed improved performance compared to the LASSO and overlapping transcripts models. Furthermore, we compared MOF detection overall versus according to subgroups of impacted organs and found no significant difference separately by any specific organ (Supplementary Table S6), suggesting that the prediction models could be useful despite some heterogeneity in the MOF outcome presentation.

**Table 5:** Sensitivity, specificity, positive predictive value (PPV) and negative predictive value (NPV) of the various models constructed

		Sensitivity	Specificity	PPV	NPV
Training set					
Models	LASSO biomarkers	0.878	0.949	0.923	0.918
	XGBoost biomarkers	0.951	1.000	1.000	0.967
	Overlapping biomarkers	0.878	0.932	0.900	0.917
	APACHEII + sex	0.341	0.746	0.483	0.620
	ISS + sex	0.268	0.695	0.379	0.577
	NISS + sex	0.293	0.780	0.480	0.613
Test set					
Models	LASSO biomarkers	0.765	0.750	0.684	0.818
	XGBoost biomarkers	0.833	0.706	0.800	0.750
	Overlapping biomarkers	0.765	0.750	0.684	0.818
	APACHEII + sex	0.353	0.708	0.462	0.607
	ISS + sex	0.353	0.750	0.500	0.621
	NISS + sex	0.235	0.750	0.400	0.581

### Molecular functions and pathways associated with the predictive biomarkers

**Fig. 3:**
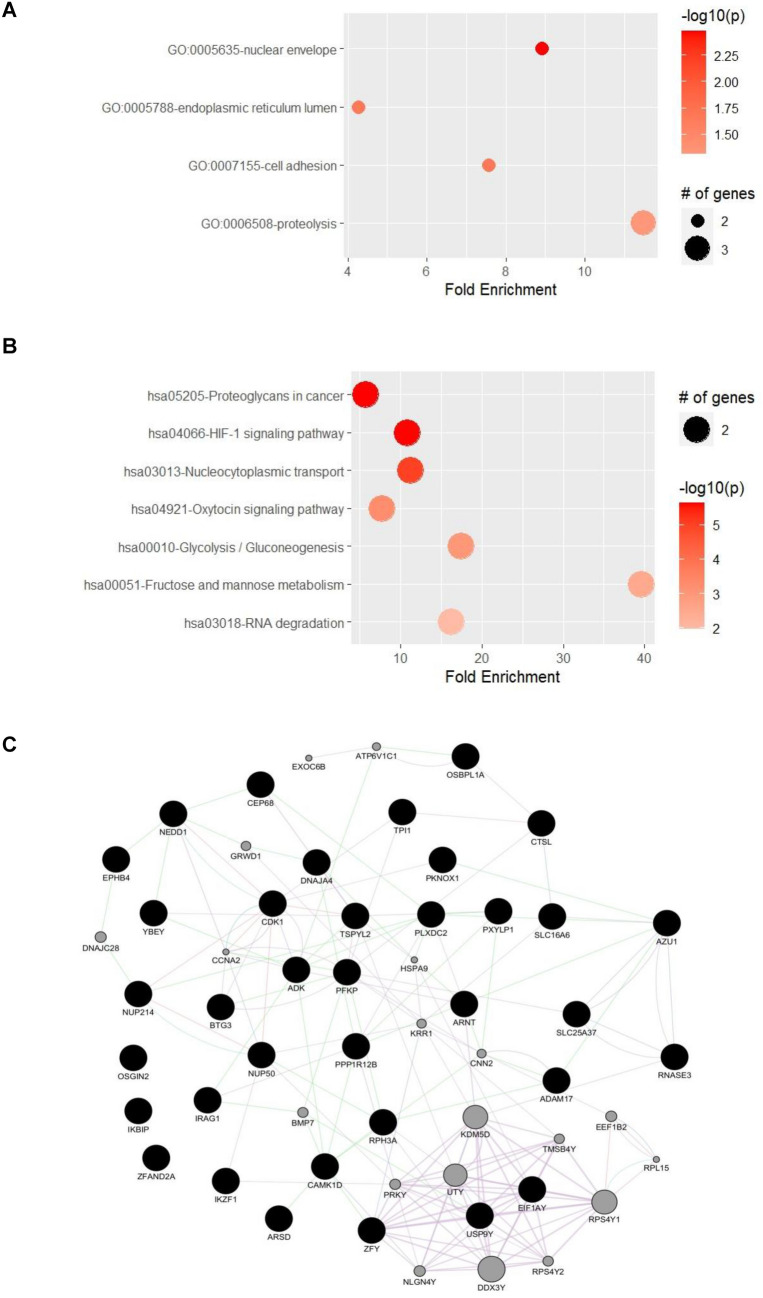
Evaluation of the molecular roles associated with the 43 predictive transcripts. Enrichment analysis evaluating **(A)** Gene Ontology (GO) and **(B)** KEGG pathway terms, and **(C)** network plot are shown

We evaluated the functional annotations of the 43 predictive transcripts identified by both models. Significant enrichment was detected for GO terms related to nuclear envelope, endoplasmic reticulum lumen, cell adhesion and proteolysis (Fig. 3A), and KEGG pathway terms related to signaling (HIF-1 and oxytocin signaling, nucleocytoplasmic transport and RNA degradation), metabolism (glycolysis/gluconeogenesis and fructose and mannose metabolism) and proteoglycans in cancer (Fig. 3B). Moreover, network analysis found that the transcripts are highly interconnected (Fig. 3C).

## Discussion

The results of our study demonstrate that indeed, MOF is a major adverse outcome among trauma patients and is strongly associated with increased mortality and prolonged length of stay. These observations support the notion that improved management of post-trauma MOF by developing better methods for early risk stratification is expected to improve patient outcomes and alleviate healthcare cost expenditure. It has been shown previously that acute organ dysfunction among sepsis patients is associated with both short-term and long-term mortality [66], which may indicate that the adverse impact of MOF could be larger than observed at the end of follow-up.

Our results show that MOF mostly occurred soon after traumatic injury, suggesting that the immune dysregulation from the impact of the trauma itself is significant, highlighting the importance of our study to analyze early (within 24 h of injury) blood molecular response to injury for subsequent outcomes. While each of the baseline injury severity scores of APACHEII, ISS and NISS tended to be higher among MOF-cases, showing that higher impact of traumatic injury may indeed render patients at increased risk, none of them were effective in MOF outcome prediction. These results support the notion that performing ML analysis of genome-wide transcriptome data to characterize patients’ responses to injury more meticulously, and using this information to develop accurate biomarker prediction models is important.

Our ML analysis identified 18 transcripts by LASSO and 41 by transcripts by XGBoost to be included in the outcome prediction model, with 16 transcripts overlapping between the two models, suggesting that they are likely important as mechanisms related to MOF. As expected, most of the transcripts have been found previously to be linked with traumatic injuries, organ failure/dysfunction and immune responses. As summarized in Table 5, various transcripts identified to be important by both LASSO and XGBoost were previously implicated in traumatic injuries and organ failure/dysfunction (*ADK*, *ARNT*/*HIF1B*, *CAMK1D*, *RPH3A*, *SLC25A37* and *ZFAND2A*) and immune cell functions (*IKZF1*, *PFKP, PKNOX1* and *TPI1*). Other transcripts have not yet been linked to traumatic injuries or inflammatory responses (*ADGRB3-*DT, CEP68, *IRAG1, PDCD4-AS1*, *YBEY* and *ZFY*), and future molecular studies to test their potential role in responses to traumatic injuries, organ failure or immunity may result in novel understanding of their mechanisms.

The proportion of males among MOF-cases was significantly higher than non-cases, which has been reported previously [67–70]. Although previous studies have suggested sex hormone signaling [70] or increased Interleukin-6 (IL-6) among males [68] as possible important molecular mechanisms, there has not yet been a study that investigated transcriptome differences, and a future study with a sufficiently large sample size to allow stratified analysis by sex is expected to be highly informative. In our study, *X-inactive specific transcript* (*XIST*), a key initiating signal for X-inactivation, was found among transcripts important for MOF outcome. *XIST* overexpression in the serum of acute pneumonia patients has been reported, showing its relevance in immune response [71]. Various studies have demonstrated that *XIST* can exert its immunomodulatory functions by binding to miRNAs and acting as a competing endogenous RNA (ceRNA)—its targets include *miR-370-3p*, a negative regulator of Toll-like receptor 4 (TLR4) [71]; *miR-132-3p*, which controls the mitogen activated protein kinase 14 (MAPK14) pathway [72]; *miR-142-5p*, which suppresses Programmed cell death protein 4 (PDCD4) [73]; and *miR-133a*, which inhibits Suppressor of cytokine signaling 2 (SOCS2) [74]. In these studies, *XIST* knockdown was found to be protective of LPS-induced apoptosis and inflammation [71]; acute lung injury [72]; acute kidney injury [73]; or myocardial ischemia reperfusion injury [74]. *XIST* inhibition was also described to mitigate sepsis-induced acute liver injury by suppressing Bromodomain-containing Protein 4 (BRD4) expression [75]. On the other hand, *XIST* was found to promote burn wound healing by suppressing *miR-19b* to enhance IL-33 expression and M2 macrophage polarization [76]. Our study also identified the importance of *USP9Y*, a male-specific transcript, previously found to be overexpressed in myocardial samples with heart failure [77]. Given our findings, further mechanistic studies to understand the impact of biological sex in MOF are expected to be highly informative.

Mortality was found only among MOF-cases, demonstrating the adverse effect of MOF. The main causes of death for these patients were attributed to MOF, or a variety of other causes, including sepsis, shock, head injury, hypoxia and brain death. It is uncertain whether the gene expression signatures this study identified to be relevant to MOF outcome may also be related to other clinical syndromes, and further assessment would be informative.

Recent studies have challenged the notion that bacterial infections are a major trigger of MOF among trauma and surgical patients, and suggested that on the other hand, MOF can also be a major contributor to infections [78]. While we found that the incidence of infections is significantly greater among MOF-cases compared to non-cases overall, MOF tended to precede infections, rather than being a consequence of them, as a previous study also reported [79]. Thus, infections appear to be both a driver and a consequence of MOF in this setting. In addition to MOF-cases, infections were also detected among a notable proportion of non-cases, consistent with previously described post-injury immune dysregulation. Trauma triggers the secretion of danger-associated molecular patterns (DAMPs) to induce a hyper-inflammatory state, termed systemic inflammatory response syndrome (SIRS) associated with early MOF, and as a counter response, also triggers the suppressing/inhibiting DAMPs (SAMPs) to induce the compensatory anti-inflammatory response syndrome (CARS), described as an immunosuppressive state that renders patients highly susceptible to nosocomial infections [9, 80–82]. In our study, we further characterized potential differences between MOF-cases and non-cases by specific infection types and microorganisms detected. Notably, we found that pneumonia incidence was higher among MOF cases compared to non-cases, with similar incidence before and after MOF. Surgical site and bloodstream infections were also more frequent among MOF cases compared to non-cases; however, the incidences increased after MOF compared to before. Interestingly, urinary tract infection incidence was lower among MOF-cases compared to non-cases, and moreover, among MOF-cases, the incidence was higher after MOF. One potential caveat is that determining infections requires swabs to be taken in a timely manner and culturing on specific selection plates, and therefore, it is possible that not all infections can be detected accurately. Nevertheless, our results suggest that further studies to characterize the timing of the association between MOF and specific types of infections are expected to provide additional insights into the mechanism of association between MOF and infections.

This study provides proof-of-concept results for the advantage of biomarker development based on ML analysis of genome-wide data to understand the molecular responses to traumatic injury that renders patients at increased risk for adverse outcomes, including MOF. The application of the results presented here in the clinical setting would be to develop a rapid assay to selectively measure the predictive transcripts from routine blood drawn at admission to the hospital, to allow calculating the predicted probability of MOF. Such a method for early patient stratification by adverse outcome risk is expected to enhance clinical decision making and expected to aid in the early implementation of surveillance and intervention strategies to mitigate the risks. As such, it may result in improving patient outcomes and alleviating healthcare costs. Early intervention strategies for patients found to be at increased risk of MOF may include immediate ICU admission, increased hemodynamic and culture monitoring, increased precision of resuscitation and more aggressive drainage of infections. Such MOF risk assessment may also be included as a factor for borderline resuscitation decisions and providing information to the patients’ family. It may also be advantageous for Crisis Standards of Care or military triage, as well as patient selection in clinical research. Given that MOF is known to be a major adverse outcome among trauma patients and is significantly associated with mortality and increased length of stay, as our results also show, novel methods to alleviate the burden of MOF are imperative.

To overcome limitations of this study, future studies to externally validate the biomarker prediction method in new large and diverse trauma patient populations, and mechanistic studies to determine how the transcripts may be related to MOF onset would strengthen our findings. Such validation and mechanistic studies may contribute to the development of novel preventative and therapeutic agents in the future.

## Conclusions

Applying ML to analyze genome-wide transcriptome data from early blood samples collected within 24 h of traumatic injury resulted in the development of an accurate prediction model for MOF based on 41 associated transcripts. The biomarker-based prediction models provided a significantly better prediction of MOF compared to those based on injury severity scores and sex (APACHEII, ISS and NISS) or the detection of infections, highlighting the importance of exploring novel molecular medicine approaches for early risk stratification.

### Supplementary Information

Additional file 1

Additional file 2

## Data Availability

The data that support the findings of this study are available from the Glue Grant Study, but restrictions apply to the availability of these data, which were used under approval for the current study, and so are not publicly available. Data are however available from the authors upon reasonable request, with permission of the Glue Grant Consortium.

## Abbreviations

ML: Machine learning
LASSO: Least absolute shrinkage and selection operator
XGBoost: EXtreme gradient boosting
APACHEII: Acute physiology and chronic health evaluation II
ISS: Injury severity score
NISS: New injury severity score
