# Supplementary material for: Development of a biomarker prediction model for post-trauma multiple organ failure/dysfunction syndrome based on the blood transcriptome
Source: Ann Intensive Care. 2024 Aug 28;14:134. doi: 10.1186/s13613-024-01364-5 (PMC11358370; doi:10.1186/s13613-024-01364-5)
Supplement: Supplementary file 1 — Additional file 1 [file 13613_2024_1364_MOESM1_ESM.docx]

|  | **All patients**  **(N=141)** | **MOF cases**  **(n=58)** | **Non-cases**  **(n=83)** | **p-val.** |
| --- | --- | --- | --- | --- |
| **INJURY MECHANISMS** | | | | |
| Fall | 9 (6.4%) | 5 (8.6%) | 4 (4.8%) | 0.781^a^ |
| Machinery | 1 (0.7%) | 1 (1.7%) | 0 (0%) |  |
| MVC – occupant | 80 (56.7%) | 33 (56.9%) | 47 (56.6%) |  |
| MVC – motorcyclist | 25 (17.7%) | 10 (17.2%) | 15 (18.1%) |  |
| MVC – cyclist | 3 (2.1%) | 1 (1.7%) | 2 (2.4%) |  |
| MVC – pedestrian | 18 (12.8%) | 6 (10.3%) | 12 (14.5%) |  |
| Struck by or against | 1 (0.7%) | 1 (1.7%) | 0 (0%) |  |
| Other | 4 (2.8%) | 1 (1.7%) | 3 (3.6%) |  |
| **INJURY CHARACTERISTICS** | | | | |
| Crush injury | 20 (13.2%) | 10 (17.2%) | 10 (12.0%) | ​​0.531^b^ |
| Severe head injury | 15 (11.2%) | 7 (12.1%) | 8 (9.6%) | 0.855^b^ |

**Supplementary Table S1. Injury mechanisms among all patients in this study.** n (%) are reported, with ^a^Fisher’s Exact two-tailed test or ^b^Chi-square test p-values.

| **Worse organ-specific score over 28 days:** | **All patients**  **(N=141)** | **MOF cases**  **(n=58)** | **Non-cases**  **(n=83)** | ***p-value*** |
| --- | --- | --- | --- | --- |
| **Central nervous system score** | 3.7 ± 0.8 | 3.8 ± 0.5 | 3.6 ± 1.0 | 0.134 |
| **Cardio score** | 2.7 ± 1.0 | 3.4 ± 0.7 | 2.1 ± 0.8 | <0.001 |
| **Respiratory score** | 1.8 ± 1.1 | 2.6 ± 0.7 | 1.3 ± 1.1 | <0.001 |
| **Renal score** | 1.0 ± 0.7 | 1.4 ± 0.9 | 0.7 ± 0.2 | <0.001 |
| **Hepatic score** | 0.7 ± 1.1 | 1.3 ± 1.3 | 0.3 ± 0.6 | <0.001 |
| **Hematologic score** | 0.6 ± 0.7 | 1.0 ± 0.8 | 0.4 ± 0.5 | <0.001 |

**Supplementary Table S2. The mean worse MOF score (out of 4) over 28 days by organ type.** Unpaired equal variance two-tailed t-test p-values are shown.

| **First infection recorded ≤ 7 days before MOF onset day** | n=6 |
| --- | --- |
| **First infection recorded on the same day MOF onset day** | n=7 |
| **First infection recorded 1-7 days after MOF onset day** | n=23 |
| **First infection recorded 8-14 days after MOF onset day** | n=9 |
| **First infection recorded 15-26 days after MOF onset day** | n=5 |
| **No MOF** | n=40 |

**Supplementary Table S3. Timing of the first recording of infection, relative to recorded MOF onset day.**

Provided as a separate table in Excel.

**Supplementary Table S4. Fold change, p-value, and FDR-adjusted p-values of the transcripts included in the prediction model.**

| **Coefficient** | **Estimate [95% CI]** | **p-val.** | **Estimate [95% CI]** | **p-val.** |
| --- | --- | --- | --- | --- |
| **LASSO model** | | | **Overlapping biomarkers model** | |
| Intercept | -0.953 [-1.857, -0.227] | 0.018 | -0.947 [-1.799, -0.236] | 0.015 |
| 1558795_at: *ADGRB3-DT* | -0.296 [-1.107, 0.566] | 0.470 | -0.305 [-1.084, 0.546] | 0.448 |
| 201037_at: *PFKP* | 0.909 [0.007, 2.108] | 0.085 | 1.034 [0.129, 2.212] | 0.049 |
| 202087_s_at: *CTSL* | -0.039 [-0.955, 0.829] | 0.931 | *Not in XGBoost model* | --- |
| 204120_s_at: *ADK* | -1.921 [-3.534, -0.861] | 0.003 | -1.804 [-3.221, -0.793] | 0.003 |
| 204195_s_at: *PKNOX1* | -1.008 [-1.913, -0.226] | 0.017 | -1.012 [-1.904, -0.244] | 0.015 |
| 205039_s_at: *IKZF1* | 0.705 [-0.308, 1.900] | 0.204 | 0.593 [-0.380, 1.729] | 0.265 |
| 205230_at: *RPH3A* | -1.006 [-1.914, -0.235] | 0.017 | -1.004 [-1.913, -0.237] | 0.017 |
| 212675_s_at: *CEP68* | 0.768 [-0.148, 1.864] | 0.125 | 0.748 [-0.108, 1.736] | 0.103 |
| 213011_s_at: *TPI1* | 0.513 [-0.530, 1.628] | 0.341 | 0.581 [-0.365, 1.634] | 0.247 |
| 214575_s_at: *AZU1* | 0.432 [-0.466, 1.416] | 0.352 | *Not in XGBoost model* | --- |
| 222307_at: *PDCD4-AS1* | -0.239 [-1.211, 0.689] | 0.613 | -0.176 [-1.090, 0.722] | 0.695 |
| 224550_s_at: *IRAG1* | -1.909 [-4.397, 0.236] | 0.095 | -1.848 [-4.126, 0.158] | 0.082 |
| 226650_at: *ZFAND2A* | 0.357 [-0.439, 1.179] | 0.377 | 0.271 [-0.482, 1.036] | 0.475 |
| 230214_at: *IRAG1* | 0.836 [-1.157, 2.950] | 0.413 | 0.750 [-1.106, 2.693] | 0.428 |
| 230619_at: *ARNT* | -1.529 [-2.820, -0.440] | 0.010 | -1.565 [-2.877, -0.481] | 0.009 |
| 230760_at: *ZFY* | 0.852 [0.166, 1.648] | 0.021 | 0.823 [0.147, 1.599] | 0.024 |
| 231078_at: *SLC25A37* | 1.051 [0.272, 2.025] | 0.016 | 1.048 [0.287, 1.975] | 0.013 |
| 235626_at: *CAMK1D* | -0.186 [-1.108, 0.703] | 0.680 | -0.215 [-1.134, 0.678] | 0.634 |
| 239208_s_at: *YBEY* | -0.299 [-1.170, 0.502] | 0.472 | -0.347 [-1.203, 0.444] | 0.398 |
| **APACHEII + sex model** | | |  |  |
| Intercept | -2.751 [-5.245, -0.482] | 0.022 |  |  |
| APACHEII | 0.064 [-0.011, 0.145] | 0.103 |  |  |
| Sex-male (ref: female) | 0.910 [0.053, 1.819] | 0.042 |  |  |
| **ISS + sex model** | | |  |  |
| Intercept | -1.691 [-3.0738, -0.422] | 0.012 |  |  |
| ISS | 0.022 [-0.008, 0.053] | 0.154 |  |  |
| Sex-male (ref: female) | 0.953 [0.088, 1.874] | 0.035 |  |  |
| **NISS + sex model** | | |  |  |
| Intercept | -1.463 [-2.960, -0.063] | 0.046 |  |  |
| NISS | 0.014 [-0.016, 0.045] | 0.371 |  |  |
| Sex-male (ref: female) | 0.897 [0.040, 1.804] | 0.045 |  |  |

**Supplementary Table S5. Coefficient estimates [95% CI] and p-values of the LASSO biomarker and clinical multivariable logistic regression models.**

| **Accuracy of detection among MOF cases (%)** | **LASSO biomarkers** | **XGBoost biomarkers** | **Overlapping biomarkers** | **APACHEII + sex** | **ISS + sex** | **NISS + sex** |
| --- | --- | --- | --- | --- | --- | --- |
| **Overall MOF cases**  **(n=58)** | 49 (84.5%) | 51 (87.9%) | 49 (84.5%) | 20 (34.5%) | 17 (29.3%) | 16 (27.6%) |
| **Organ-specific score ≥2 recorded**  **(n among MOF cases)** |  |  |  |  |  |  |
| **Cardio**  **(n=55)** | 46 (83.6%)  p=0.852 | 49 (89.1%)  p=1.000 | 46 (83.6%)  p=0.852 | 20 (36.4%)  p=0.778 | 17 (30.9%)  p=0.769 | 16 (29.1%)  p=0.765 |
| **Respiratory**  **(n=47)** | 40 (85.1%)  p=1.000 | 45 (95.7%)  p=0.117 | 40 (85.1%)  p=1.000 | 18 (38.3%)  p=0.646 | 15 (31.9%)  p=0.749 | 14 (29.8%)  p=0.745 |
| **Renal**  **(n=12)** | 10 (83.3%)  p=1.000 | 12 (100%)  p=0.383 | 10 (83.3%)  p=1.000 | 5 (41.7%)  p=0.562 | 4 (33.3%)  p=0.756 | 5 (41.7%)  p=0.331 |
| **Hepatic**  **(n=17)** | 17 (100%)  p=0.094 | 16 (94.1%)  p=0.712 | 17 (100%)  p=0.094 | 6 (35.3%)  p=1.000 | 4 (23.5%)  p=0.792 | 4 (23.5%)  p=1.000 |
| **Hematologic**  **(n=6)** | 6 (100%)  p=0.600 | 6 (100%)  p=1.000 | 6 (100%)  p=0.600 | 3 (50%)  p=0.422 | 1 (16.7%)  p=0.678 | 2 (33.3%)  p=0.671 |

**Supplementary Table S6. Accuracy of MOF outcome prediction among cases, overall and separately by worst organ-specific score ≥2 out of 4 recorded at any time over 28 days.** All MOF patients had a record of worse central nervous system score ≥2 and variable recorded specific organ scores ≥2, as shown above. The counts and proportions of correctly identified cases are shown separately by each impacted organ, with two-sided exact binomial test p-values compared to the expected proportions according to overall MOF prediction for each model.
